# Supplementary material for: Calculation of steel corrosion rate of reinforced concrete slab based on rust expansion crack
Source: PLoS One. 2025 May 12;20(5):e0322344. doi: 10.1371/journal.pone.0322344 (PMC12068711; doi:10.1371/journal.pone.0322344)
Supplement: S1 Table — (DOCX) [file pone.0322344.s001.docx]

**S1 Table. Effects of protective layer thickness (I# slab)**

| Rebar  number | Rebar  length/(mm) | Rebar  diameter/(mm) | Uncorroded  weight/（kg） | Corroded  weight/（kg） | Corresponding protective layer thickness/（mm） | Amount of rebar corrosion/（%） | | Width of  cracks /(mm) | |
| --- | --- | --- | --- | --- | --- | --- | --- | --- | --- |
| 1 | 500 | 25 | 1.716 | 1.688 | **30** | 1.63 | **1.63**  **（avg）** | 0.21 | **0.205**  **(avg)** |
| 2 | 500 | 25 | 1.721 | 1.693 |  | 1.63 |  | 0.20 |  |
| 3 | 500 | 25 | 1.721 | 1.692 | **40** | 1.66 | **1.46**  **(avg)** | 0.24 | **0.185**  **(avg)** |
| 4 | 500 | 25 | 1.716 | 1.695 |  | 1.26 |  | 0.13 |  |
| 5 | 500 | 25 | 1.712 | 1.698 | **50** | 0.79 | **1.225**  **(avg)** | 0.11 | **0.155**  **(avg)** |
| 6 | 500 | 25 | 1.719 | 1.691 |  | 1.66 |  | 0.20 |  |
