# Supplementary material for: Calculation of steel corrosion rate of reinforced concrete slab based on rust expansion crack
Source: PLoS One. 2025 May 12;20(5):e0322344. doi: 10.1371/journal.pone.0322344 (PMC12068711; doi:10.1371/journal.pone.0322344)
Supplement: S2 Table — (DOCX) [file pone.0322344.s002.docx]

**S2 Table. Influence of spacing between distributed reinforcement** **(II# slab)**

| Rebar  number | Rebar  length/(mm) | Rebar  diameter/(mm) | Uncorroded  weight/(kg) | Corroded  weight/(kg) | Corresponding protective layer thickness/(mm) | Diameter  of distributed rebar/(mm) | Spacing of distributed rebar/(mm) | Amount of rebar corrosion/(%) | | Width of  crack/(mm) | |
| --- | --- | --- | --- | --- | --- | --- | --- | --- | --- | --- | --- |
| 7 | 1000 | 25 | 3.433 | 3.415 | 40 | 10 | **100** | 0.53 | **0.54**  **(avg)** | 0.11 | **0.115**  **(Avg)** |
| 8 | 1000 | 25 | 3.434 | 3.416 | 40 | 10 |  | 0.55 |  | 0.12 |  |
| 9 | 1000 | 25 | 3.433 | 3.412 | 40 | 10 | **200** | 0.60 | **0.65**  **(avg)** | 0.15 | **0.165**  **(Avg)** |
| 10 | 1000 | 25 | 3.440 | 3.461 | 40 | 10 |  | 0.70 |  | 0.18 |  |
