# Supplementary material for: Calculation of steel corrosion rate of reinforced concrete slab based on rust expansion crack
Source: PLoS One. 2025 May 12;20(5):e0322344. doi: 10.1371/journal.pone.0322344 (PMC12068711; doi:10.1371/journal.pone.0322344)
Supplement: S3 Table — (DOCX) [file pone.0322344.s003.docx]

**S3 Table. Analysis of the model calculated values and test data values of I# slab and II# slab**

| I# slab | | | | II# slab | | | |
| --- | --- | --- | --- | --- | --- | --- | --- |
| Rebar number | Measured value (%) | Calculated value (%) | Deviation  (%) | Rebar number | Measured value (%) | Calculated value (%) | Deviation  (%) |
| 1 | 1.63 | 1.52 | 6.75 | 7 | 0.53 | 0.48 | 9.43 |
| 2 | 1.63 | 1.50 | 7.98 |  |  |  |  |
|  |  |  |  | 8 | 0.55 | 0.49 | 10.91 |
| 3 | 1.66 | 1.56 | 6.02 |  |  |  |  |
| 4 | 1.26 | 1.11 | 11.90 | 9 | 0.60 | 0.53 | 11.67 |
| 5 | 0.79 | 0.72 | 8.86 |  |  |  |  |
|  |  |  |  | 10 | 0.70 | 0.64 | 8.57 |
| 6 | 1.66 | 1.54 | 7.23 |  |  |  |  |
