# Supplementary figures and images for: Calculation of steel corrosion rate of reinforced concrete slab based on rust expansion crack
Source: PLoS One. 2025 May 12;20(5):e0322344. doi: 10.1371/journal.pone.0322344 (PMC12068711; doi:10.1371/journal.pone.0322344)

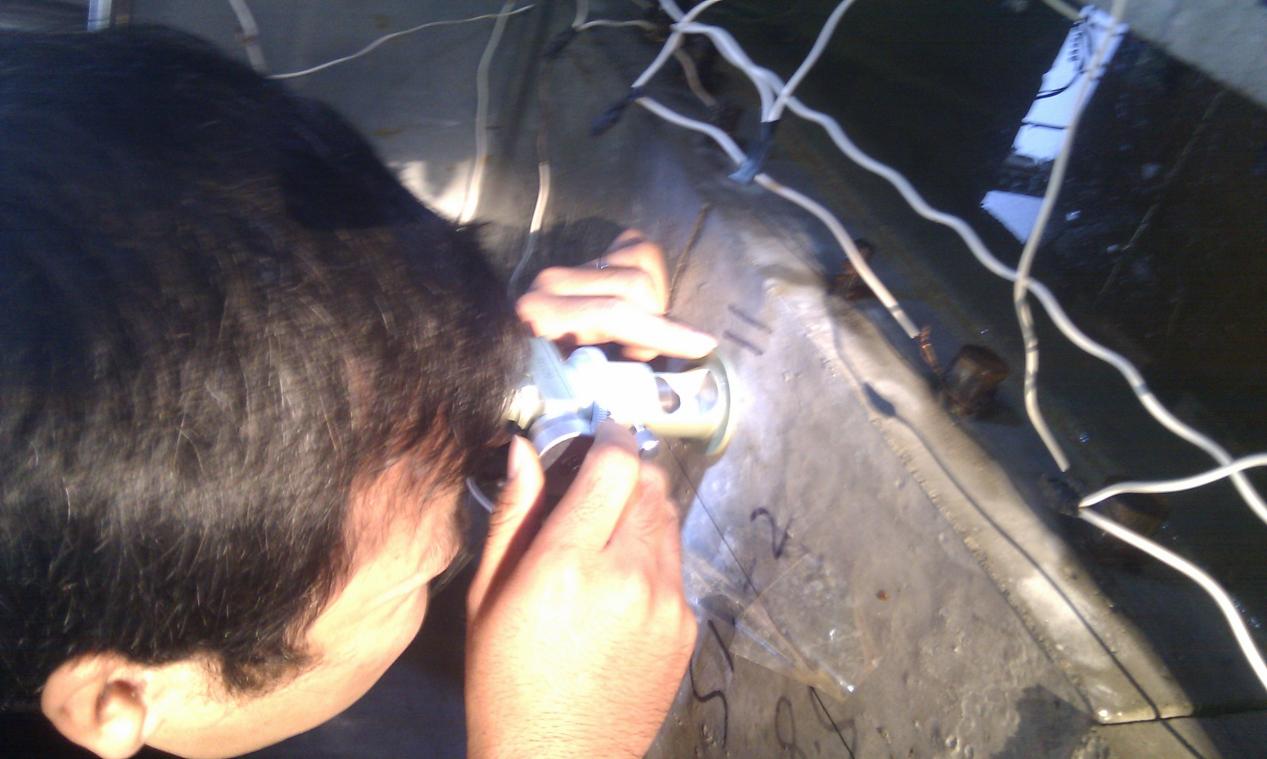


**S1 Figure.** Crack width test on board surface

Supplement: S1 Figure — (DOCX) [file pone.0322344.s004.docx]
