# Supplementary material for: Calculation of steel corrosion rate of reinforced concrete slab based on rust expansion crack
Source: PLoS One. 2025 May 12;20(5):e0322344. doi: 10.1371/journal.pone.0322344 (PMC12068711; doi:10.1371/journal.pone.0322344)
Supplement: Attachment — Submitted filename: PlosOne2025.pdf [file pone.0322344.s005.pdf]

Dear authors,

The work is interesting and deserves to be published after significant revisions. My suggestions have been made based on each part of your manuscript's structure.

Introduction:

1. **Stronger Contextualization**:
   - Begin the introduction with an impactful statement that links the topic to broader challenges in civil engineering. For example:
     - "Ensuring the durability and safety of reinforced concrete structures has become a critical focus in civil engineering, particularly in the context of increasing environmental challenges and aging infrastructure."
2. **Clear Problem and Gap Statement**:
   - Clearly articulate the gap this study addresses. For example:
     - "While significant progress has been made in understanding the relationship between corrosion and crack propagation in concrete structures, existing models often neglect the influence of distributed reinforcement, which plays a crucial role in practical scenarios. This gap limits the applicability of these models in real-world conditions."
3. **Refined Objective Statement**:
   - Rewrite the objective to emphasize its scientific and practical impact. For example:
     - "This study aims to develop a refined calculation model that incorporates the effects of distributed reinforcement on crack propagation, validated through experimental and theoretical analysis, to improve the accuracy and applicability of non-destructive corrosion assessments."
4. **List of Contributions**:
   - Add a paragraph explicitly outlining the study's contributions, such as:
     - "The key contributions of this study include:

        - Developing a novel calculation model that accounts for distributed reinforcement.
        - Conducting experimental validation to ensure the model's practical reliability.
        - Providing insights for non-destructive testing methods applicable to real-world engineering."

5. **Improved Style and Cohesion**:
    - Simplify overly technical sentences and ensure clarity for an interdisciplinary audience. For instance, break down longer sentences into smaller, more digestible ones. For example:
        - Replace: "The test method of accelerating the surface cracking of reinforced concrete slabs with reinforcement electrification was used to test the relationship between the surface crack width of reinforced concrete slabs and the corrosion rate of main reinforcement."
        - With: "An accelerated surface cracking method was used, employing reinforcement electrification. This approach tested the relationship between crack width in reinforced concrete slabs and the corrosion rate of main reinforcement."
6. **Incorporate a Relevant Study**:
    - Reference and integrate insights from a recent, relevant study, such as the MDPI article *"Corrosion Monitoring Techniques in Reinforced Concrete Structures: A Review"* (https://www.mdpi.com/2075-4701/12/3/417). For example:
        - "Corrosion in reinforced concrete structures is a critical challenge requiring precise detection and analysis. Recent advancements in corrosion monitoring techniques, as reviewed by [Author(s), 2022] in *Metals*, emphasize the growing application of non-destructive methods such as acoustic emission and electrochemical techniques for real-time corrosion assessment. This study builds on these advancements by integrating the role of distributed reinforcement into the analysis of crack behavior."

"Modeling" section

1. Clarify Objectives

- The objectives of the model are presented, but they could be better emphasized. For example:
    - Explicitly state that the model is intended to link crack width with reinforcement corrosion and assess its predictive power compared to experimental data.

2. Contextualize the Model's Novelty

- Highlight the novelty of the proposed model compared to previous works. For example:
    - "Unlike previous models [References], this study incorporates distributed reinforcement and its early corrosion effects, providing a more comprehensive framework for assessing crack propagation and steel corrosion in reinforced concrete."

3. Explain Key Equations

- Some equations (e.g., Equation 1 and 2) could benefit from more intuitive explanations or physical interpretations. For instance:
    - Discuss how each parameter (e.g., stiffness reduction factor, distributed reinforcement spacing) impacts the model predictions.

4. Integrate Practical Implications

- Emphasize how the model can be applied in engineering practice. For example:
    - "This model enables engineers to predict crack widths and corrosion rates, aiding in proactive maintenance and extending the lifespan of critical infrastructure."

5. Incorporate Figures and Diagrams

- The section already includes schematics of slabs and equations, but diagrams explaining the physical phenomena (e.g., crack propagation with and without distributed reinforcement) could help visually clarify key concepts.

6. Compare with Existing Models

- Add a paragraph comparing your model to widely cited models (e.g., Li-CQ's model). Highlight improvements, such as:
    - Reduced computational complexity.
    - Better alignment with experimental results under practical conditions.

7. Address Limitations

- Briefly discuss limitations and assumptions, such as:
    - Neglecting the effects of external stresses or environmental variability.
    - Using a simplified corrosion process in calculations.

Example Integration with Context from the Paper:

- "Building on the work of Chen et al. [34], this study advances the understanding of distributed reinforcement by introducing an adjusted stiffness reduction factor and experimental validation. For instance, the presented Equation (3) refines the crack-width prediction, accommodating nonlinear effects observed during accelerated corrosion tests."

<u>Experiment section</u>

1. Clarify the Experiment Objectives

- Reinforce the main objectives and the rationale for choosing the experimental variables at the beginning of the section. For example:
  - "The primary objective of the experimental setup is to validate the proposed model by analyzing the relationship between surface crack width and reinforcement corrosion under different protective layer thicknesses and distributed reinforcement configurations."

2. Enhance the Procedure Description

- Ensure the procedure is described clearly enough for replication. Consider detailing:
  - Why the semi-wet electrification method was chosen for accelerated corrosion.
  - Justify the use of a 5% NaCl solution and how it simulates real-world conditions.

3. Organize Data and Results

- Ensure that the experimental data (tables and figures) are clearly linked to the hypotheses and objectives. Add more detailed descriptions, such as:
  - "Table 1 highlights the correlation between protective layer thickness and reinforcement corrosion, indicating that thicker layers effectively reduce the corrosion rate and crack width."

4. Discuss Experimental Limitations

- Include a brief paragraph about potential limitations of the experiment. For example:
  - "The accelerated corrosion method may not fully replicate the long-term effects of natural environmental conditions on concrete structures, potentially limiting the generalizability of the findings."

5. Explore Comparisons Between Samples

- Include direct comparisons between the I# and II# slabs, emphasizing differences in corrosion and crack patterns. For example:
  - "The presence of distributed reinforcement in the II# slab reduced the overall corrosion rate by 55.5% compared to the I# slab, as shown in Table 2. This demonstrates the effectiveness of distributed reinforcement in mitigating crack development."

6. Integrate Discussions of Figures and Tables

- Ensure that figures and tables are explicitly discussed in the text. For example:
  - "Figure 10 illustrates the accelerated corrosion setup, which ensures uniform NaCl distribution and maintains consistent semi-wet conditions. Observations from this setup, such as bubble formation, confirm the ongoing corrosion process."

7. Link Results to the Model

- Relate the experimental findings directly to the proposed model. For example:
  - "The experimental findings in Table 2 align with the predictions of Equation (3), particularly in cases where distributed reinforcement spacing was 100 mm."

8. Add Practical Context

- Discuss how the experimental results can be applied in practice. For example:

    - "These experimental insights provide a framework for engineers to design reinforced concrete structures with optimized crack control, thereby extending their service life."

Suggested Introduction for the Section:

- "The experimental setup was designed to investigate the effects of protective layer thickness and distributed reinforcement spacing on crack width and reinforcement corrosion. Using a semi-wet electrification method, the study aimed to replicate accelerated corrosion conditions while maintaining relevance to practical engineering scenarios."

Results of the Experiment

1. Highlight Key Findings

- Start with a concise summary of the main findings. For example:
  - "The results demonstrate a clear relationship between protective layer thickness, distributed reinforcement spacing, and the extent of reinforcement corrosion and surface crack width."

2. Organize Results Logically

- Structure the results by grouping them into key themes, such as:
  - Effects of protective layer thickness (e.g., as in Table 1).
  - Influence of distributed reinforcement (e.g., as in Table 2).

3. Interpret Data Clearly

- Provide clear interpretations of the data presented in tables and figures. For example:
  - "As shown in Table 1, the corrosion rate decreases as the protective layer thickness increases, reducing crack width by an average of 25% between 30 mm and 50 mm layers."

4. Discuss Trends and Correlations

- Identify and explain notable trends or correlations. For example:
  - "The results in Table 2 indicate that reducing the spacing of distributed reinforcement from 200 mm to 100 mm decreases the corrosion rate by 17%, suggesting that closer reinforcement spacing improves structural durability."

5. Compare Experimental and Theoretical Results

- Discuss how the experimental data compares to the theoretical model. For example:
    - "Figures 11 and 12 illustrate that the theoretical model predicts corrosion rates with a deviation of 10–15% for slabs without distributed reinforcement and 8–13% for slabs with distributed reinforcement, confirming the model's reliability."

6. Include Practical Implications

- Relate the findings to real-world applications. For example:
    - "These findings suggest that increasing protective layer thickness and optimizing distributed reinforcement spacing can significantly enhance the durability of reinforced concrete structures exposed to chloride environments."

7. Address Limitations or Variability

- Discuss any variability in the results and potential limitations. For example:
    - "Variations in corrosion rates between samples may be attributed to inconsistencies in the curing process or differences in the distribution of the NaCl solution during testing."

8. Add Visual Clarity

- Ensure that figures and tables are clearly labeled and referenced in the text. Use captions to summarize key takeaways for each figure/table.

Suggested Section Start:

- "The experimental results reveal critical insights into the effects of protective layer thickness and distributed reinforcement on reinforcement corrosion and surface crack width. Tables 1 and 2 summarize the test data, while Figures 11 and 12 compare experimental values with theoretical predictions."

Modeling Verification section:

1. Clarify the Purpose of the Verification

- Begin the section by stating why the modeling verification is critical. For example:
    - "This section validates the accuracy and applicability of the proposed model by comparing theoretical predictions with experimental data and practical engineering results."

2. Organize the Verification Process

- Divide the section into logical subsections, such as:
    1. **Experimental Verification**: Comparison between experimental results and theoretical predictions.
    2. **Practical Engineering Verification**: Application of the model to real-world cases.

3. Emphasize Comparisons

- Highlight the comparison between experimental and theoretical results. For example:
    - "As shown in Figures 11 and 12, the proposed model predicts corrosion rates with deviations ranging from 10% to 15% for slabs without distributed reinforcement and 8% to 13% for slabs with distributed reinforcement, demonstrating its reliability and accuracy."

4. Discuss Real-World Applications

- For practical engineering verification, describe how the model was applied to the Sanhongqi Overpass. For example:
  - "The model was applied to the Sanhongqi Overpass, where the theoretical corrosion rate was calculated as 9.20%, compared to the measured value of 10.56%, with a deviation of 12.88%. This result confirms the model's applicability to real-world scenarios."

5. Include Practical Recommendations

- Provide recommendations for using the model in practical engineering. For example:
  - "To improve accuracy in practical applications, a correction factor between 1.05 and 1.15 is recommended, based on the specific design parameters of the structure."

6. Discuss Limitations and Future Refinements

- Acknowledge the limitations of the verification process. For example:
  - "While the model shows high accuracy in predicting corrosion rates, factors such as environmental variability and external stresses were not considered and should be addressed in future studies."

7. Visual Representation of Results

- Use charts and graphs effectively. For example:
  - Ensure Figures 11 and 12 are referenced directly in the discussion with clear explanations of what they illustrate about the model's accuracy.

Suggested Start of the Section:

- "The proposed model was verified through two approaches: comparison with experimental data and application to a real-world engineering case. The experimental results confirmed the model's accuracy within an acceptable deviation range, while the practical application demonstrated its relevance to engineering practice."

Conclusion section:

1. Summarize Key Findings

- Start with a concise summary of the main findings from the study. For example:
  - "This study developed a theoretical model linking surface crack width and reinforcement corrosion in reinforced concrete slabs. The model accounts for the effects of distributed reinforcement and protective layer thickness, providing accurate predictions validated by experimental data and real-world applications."

2. Emphasize Contributions

- Clearly outline the contributions of the research. For example:
  - "The key contributions of this work include:
    1. Introducing a modified stiffness reduction factor for improved prediction accuracy.
    2. Demonstrating the significant role of distributed reinforcement in reducing corrosion rates and crack propagation.
    3. Providing practical recommendations for optimizing structural durability in chloride environments."

3. Highlight Practical Implications

- Relate the findings to practical engineering applications. For example:
  - "The proposed model offers a valuable tool for engineers to assess and mitigate corrosion in reinforced concrete structures, supporting proactive maintenance and extending service life."

4. Acknowledge Limitations

- Briefly mention the study's limitations and areas for improvement. For example:
  - "While the model demonstrates high accuracy, factors such as environmental variability and the effects of external loads were not addressed and should be explored in future research."

5. Provide Future Directions

- Suggest avenues for future research. For example:
  - "Future studies could focus on integrating additional variables, such as long-term environmental effects and multi-directional stress states, to further enhance the model's applicability."

Suggested Start for the Conclusion:

- "This study presents a comprehensive analysis of the relationship between surface crack width and reinforcement corrosion in reinforced concrete slabs. By incorporating the influence of distributed reinforcement and protective layer thickness, the proposed model achieves high predictive accuracy, as confirmed by experimental and practical validation."

6. End with Impact

- Conclude with a statement on the broader impact of the research. For example:
  - "These findings contribute to advancing non-destructive testing techniques and improving the design and maintenance of reinforced concrete structures, addressing critical challenges in modern civil engineering."
