# Supplementary material for: Calculation of steel corrosion rate of reinforced concrete slab based on rust expansion crack
Source: PLoS One. 2025 May 12;20(5):e0322344. doi: 10.1371/journal.pone.0322344 (PMC12068711; doi:10.1371/journal.pone.0322344)
Supplement: Attachment — Submitted filename: Response to reviewers.docx [file pone.0322344.s007.docx]

Responses to Reviewer 1

Thank you for reviewing our manuscript and for your valuable comments. We take these suggestions very seriously and have revised the manuscript accordingly based on your feedback. The following is our itemized response to the review comments.

**A stronger contextualization and clearer problem statement in the introduction.**

**1. Begin the introduction with an impactful statement that links the topic to broader challenges in civil engineering.**

**Reply:** Thank you for your valuable suggestions on the introduction section of our manuscript. At the begin the introduction, we have added an impactful statement that links the topic to broader challenges in civil engineering: “Ensuring the durability and safety of reinforced concrete structures has become a critical focus in civil engineering, particularly in the context of increasing environmental challenges and aging infrastructure.With the long-term application of engineering structures,”

2. **Clearly articulate the gap this study addresses.**

**Reply:** Thank you for your valuable suggestions on the introduction section. While significant progress has been made in understanding the relationship between corrosion and crack propagation in concrete structures, existing models [12-17] often neglect the influence of distributed reinforcement, which plays a crucial role in practical scenarios. This gap limits the applicability of these models in real world conditions.

**3. Rewrite the objective to emphasize its scientific and practical impact.**

**Reply:** Thank you very much for your suggestion. To emphasize its scientific and practical impact, we have rewritten the objective to replace “The width of concrete surface crack is one of the intuitive methods to judge the corrosion degree of steel bars in concrete, and then to evaluate the durability of reinforced concrete structures. ” with “This study aims to develop a refined calculation model that incorporates the effects of distributed reinforcement on crack propagation, validated through experimental and theoretical analysis, to improve the accuracy and applicability of nondestructive corrosion assessments.” .

**4. Add a paragraph explicitly outlining the study’s contributions.**

**Reply:** Thank you for your valuable suggestion regarding the the study’s contributions of the manuscript. The key contributions of this study include: 1) developing a novel calculation model that accounts for distributed reinforcement, 2) conducting experimental validation to ensure the model's practical reliability, and 3) providing insights for non-destructive testing methods applicable to real-world engineering.

5. **Simplify overly technical sentences and ensure clarity for an interdisciplinary audience. For instance, break down longer sentences into smaller, more digestible ones.**

**Reply:** Thank you for your valuable suggestions. We have replaced “The test method of accelerating the surface cracking of reinforced concrete slabs with reinforcement electrification was used to test the relationship between the surface crack width of reinforced concrete slabs and the corrosion rate of main reinforcement.” with “An accelerated surface cracking method was used, employing reinforcement electrification. This approach tested the relationship between crack width in reinforced concrete slabs and the corrosion rate of main reinforcement.”

6. **Reference and integrate insights from a recent, relevant study.**

**Reply:** Thank you for your valuable suggestions on the introduction section, and for providing the literature should be added to the article. Corrosion in reinforced concrete structures is a critical challenge requiring precise detection and analysis. Recent advancements in corrosion monitoring techniques, as reviewed by Meyer et al [3], emphasize the growing application of non-destructive methods such as acoustic emission and electrochemical techniques for real-time corrosion assessment. This study builds on these advancements by integrating the role of distributed reinforcement into the analysis of crack behavior.

Reference

[3] Meyer Y A, Menezes I, Bonatti R S, et al. EIS Investigation of the Corrosion Behavior of Steel Bars Embedded into Modified Concretes with Eggshell Contents. Metals. 2022; 12(3):417.

**Improved clarity and emphasis on the novelty of the proposed model in the modeling section.**

**1. The objectives of the model are presented, but they could be better emphasized.**

**Reply:** We agree with the reviewer. It is explicitly state that the model is intended to link crack width with reinforcement corrosion and assess its predictive power compared to experimental data.

**2. Highlight the novelty of the proposed model compared to previous works.**

**Reply:** Thank you very much for your suggestion. We have highlighted the novelty of the proposed model compared to previous works. Unlike previous models [12-17], this study incorporates distributed reinforcement and its early corrosion effects, providing a more comprehensive framework for assessing crack propagation and steel corrosion in reinforced concrete.

3. **Some equations (e.g., Equation 1 and 2) could benefit from more intuitive explanations or physical interpretations.**

**Reply:** Thank you for your suggestion. We have conducted a detailed analysis and description of the physical meanings of the relevant parameters in equations in the revised manuscript. From Equation (1) and (2), it can be seen that the stiffness degradation coefficient of concrete increases with the increase of corrosion time, and the accumulation of corrosion products is directly proportional to the width of surface cracks in the component.

**4. Emphasize how the model can be applied in engineering practice.**

**Reply:** Thank you for your feedback and valuable comments on our manuscript. We have emphasized how the model can be applied in engineering practice. This allows engineers to predict crack width and corrosion rate, which helps to proactively maintain and extend the life of critical infrastructure.

**5. The section already includes schematics of slabs and equations, but diagrams explaining the physical phenomena (e.g., crack propagation with and without distributed reinforcement) could help visually clarify key concepts.**

**Reply:** Thank you for your suggestion. In revised manuscript, we have added the influence of different protective layer thicknesses on crack development, as shown in Figure 2.

**Figure 2.** **The influence of different protective layer thicknesses on crack development**

**6.Add a paragraph comparing your model to widely cited models (e.g., Li-CQ’s model).**

**Reply:** Thank you for your valuable comments on this study. Compared with widely used models [12-17], the model established in this paper that considers the influence of distributed steel bars not only reduces computational difficulty, but also has better alignment with experimental results under practical conditions. It should be pointed out that the calculation model obtained in this study can better reflect the corrosion mechanism of steel bars in actual engineering components.

7. **Briefly discuss limitations and assumptions.**

**Reply:** Thank you for your suggestion. We have already explained the limitations and assumptions of the proposed model in the end of Modeling section. It is worth pointing out that although this article considers the influence of distributed steel bars in actual components on the corrosion process of stressed steel bars, there are still certain shortcomings, such as neglecting the effects of external stresses or environmental variability, and using a simplified corrosion process in calculations.

8. **Example Integration with Context from the Paper.**

**Reply:** Thank you for your careful review and valuable comments on our manuscript. We have integrated with context from the paper. Building on the work of Chen et al. [35], this study advances the understanding of distributed reinforcement by introducing an adjusted stiffness reduction factor and experimental validation. For instance, the presented Equation (3) refines the crack-width prediction, accommodating nonlinear effects observed during accelerated corrosion tests.

**Detailed procedural descriptions and connections to practical engineering in the experiment section.**

1. **Reinforce the main objectives and the rationale for choosing the experimental variables at the beginning of the section.**

**Reply:** Thank you for your valuable comments on our manuscript. We have added the the main objectives and the rationale for choosing the experimental variables at the beginning of the Experiment section. The primary objective of the experimental setup is to validate the proposed model by analyzing the relationship between surface crack width and reinforcement corrosion under different protective layer thicknesses and distributed reinforcement configurations.

**2.Ensure the procedure is described clearly enough for replication. Consider detailing:Why the semi-wet electrification method was chosen for accelerated corrosion. Justify the use of a 5% NaCl solution and how it simulates real-world conditions.**

**Reply:** Thank you for your valuable comments on our manuscript. The accelerated corrosion test of steel bars in concrete specimens is generally divided into three categories: full-immersion electrification method, veneer electrification method, and semi-wet electrification method. Among them, the advantage of full-immersion electrification method is that it has a fast corrosion rate, but it requires higher testing equipment. Moreover, due to the lower oxygen content in water compared to air, the generated corrosion products are also relatively simple. The advantage of the veneer electrification method is that it can be used on site and for large components, but the component production time is relatively long and the economy is poor. The advantage of the semi-wet electrification method is that it is suitable for most components, and the corrosion products also have similar natural conditions, but the disadvantage is that the amount and concentration of electrolyte need to be observed. In view of this, this article selected a semi-wet electrification method for accelerated corrosion testing of steel bars.

This article conducted actual measurements on the corrosion law of steel bars in the Sanhongqi Bridge, which is located in a seawater environment with a NaCl solution concentration of approximately 5%. To simulate real-world conditions, the NaCl solution concentration selected in this article is 5%.

**3. Ensure that the experimental data (tables and figures) are clearly linked to the hypotheses and objectives.**

**Reply:** Thank you for your suggestion. Table 1 highlights the correlation between protective layer thickness and reinforcement corrosion, indicating that thicker layers effectively reduce the corrosion rate and crack width.

**4. Include a brief paragraph about potential limitations of the experiment.**

**Reply:** Thanks for the suggestion, here is the potential limitations of the experiment of my changes. Although accelerated corrosion methods may not fully replicate the long-term effects of natural environmental conditions on concrete structures, limiting the generalizability of research results.

5. **Include direct comparisons between the I# and II# slabs, emphasizing differences in corrosion and crack patterns.**

**Reply:** Thanks for your advice. The presence of distributed reinforcement in the II# slab reduced the overall corrosion rate by 55.5% compared to the I# slab, as shown in Table 2. This demonstrates the effectiveness of distributed reinforcement in mitigating crack development.

**6. Ensure that figures and tables are explicitly discussed in the text.**

**Reply:**Thank you for your careful review of the manuscript and your valuable suggestions. Figure 10 illustrates the accelerated corrosion setup, which ensures uniform NaCl distribution and maintains consistent semi-wet conditions. Observations from this setup, such as bubble formation, confirm the ongoing corrosion process.

**7. Relate the experimental findings directly to the proposed model.**

**Reply:** Thank you for your valuable comments on our manuscript. The experimental findings in Table 2 align with the predictions of Equation (3), particularly in cases where distributed reinforcement spacing was 100 mm.

**8. Discuss how the experimental results can be applied in practice.**

**Reply:** Thank you for your suggestion. These experimental insights provide a framework for engineers to design reinforced concrete structures with optimized crack control, thereby extending their service life.

**9. Suggested Introduction for the Section.**

**Reply:** Thank you for your careful review and suggestions regarding our manuscript. The experimental setup was designed to investigate the effects of protective layer thickness and distributed reinforcement spacing on crack width and reinforcement corrosion. Using a semi-wet electrification method, the study aimed to replicate accelerated corrosion conditions while maintaining relevance to practical engineering scenarios.

**Results of the Experiment.**

**1. Start with a concise summary of the main findings.**

**Reply:** Thank you for your valuable suggestions. The results demonstrate a clear relationship between protective layer thickness, distributed reinforcement spacing, and the extent of reinforcement corrosion and surface crack width.

**2. Structure the results by grouping them into key themes.**

**Reply:** Thank you again for your rigorous reviewing attitude. We have changed the titles of Tables 1 and 2 to "Effects of protective layer thickness" and "Influence of spacing between distributed reinforcement" to reflect the effects of protective layer thickness and spacing between distributed reinforcement, respectively.

**3. Provide clear interpretations of the data presented in tables and figures.**

**Reply:** We agree with the reviewer. As shown in Table 1, the corrosion rate decreases as the protective layer thickness increases, reducing crack width by an average of 25% between 30 mm and 50 mm layers.

**4. Identify and explain notable trends or correlations.**

**Reply:** We agree with the reviewer. The results in Table 2 indicate that reducing the spacing of distributed reinforcement from 200 mm to 100 mm decreases the corrosion rate by 17%, suggesting that closer reinforcement spacing improves structural durability.

**5. Discuss how the experimental data compares to the theoretical model.**

**Reply:** Thanks for the reviewer's suggestion. Table3, Figures 12 and 13 illustrate that the theoretical model predicts corrosion rates with a deviation of 6% to 12% for slabs without distributed reinforcement and 8% to 12% for slabs with distributed reinforcement, confirming the model's reliability.

**6. Relate the findings to real-world applications.**

**Reply:** Thank you for your careful review and valuable comments on our manuscripts. These findings suggest that increasing protective layer thickness and optimizing distributed reinforcement spacing can significantly enhance the durability of reinforced concrete structures exposed to chloride environments.

**7. Discuss any variability in the results and potential limitations.**

**Reply:** Thank you for your careful review and valuable comments on our manuscripts. It should be noted that variations in corrosion rates between samples may be attributed to inconsistencies in the curing process or differences in the distribution of the NaCl solution during testing.

**8. Ensure that figures and tables are clearly labeled and referenced in the text. Use captions to summarize key takeaways for each figure/table.**

**Reply:** Thank you for your careful review of the manuscript. We have modified the titles of Table 1, Table 2, Figure 12, and Figure 13 to ensure that figures and tables are clearly labeled and referenced in the text.

**9. Suggested Section Start.**

**Reply:** Thank you for your suggestion. The experimental results reveal critical insights into the effects of protective layer thickness and distributed reinforcement on reinforcement corrosion and surface crack width. Tables 1 and 2 summarize the test data, while Table 3, Figures 12 and 13 compare experimental values with theoretical predictions.

**Modeling Verification section.**

**1. Begin the section by stating why the modeling verification is critical.**

**Reply:** Thank you for your valuable comments on our manuscript. This section validates the accuracy and applicability of the proposed model by comparing theoretical predictions with experimental data and practical engineering results.

**2. Divide the section into logical subsections.**

**Reply:** Thank you for your careful review of the logicality of the manuscript. We have replaced the subsection “4.1 Validate by experiments” with “4.1 Experimental Verification” to confirm the comparison between experimental results and theoretical predictions, and replaced subsection “4.2 Validate by practical engineering ” with “4.2 Practical Engineering Verification” to approve the application of the model to realworld cases.

**3. Highlight the comparison between experimental and theoretical results.**

**Reply:** Thank you for your careful review and valuable comments on our manuscripts. As shown in Table 3, Figures 12 and 13, the proposed model predicts corrosion rates with deviations ranging from 6% to 12% for slabs without distributed reinforcement and 8% to 12% for slabs with distributed reinforcement, demonstrating its reliability and accuracy.

**4. For practical engineering verification, describe how the model was applied to the Sanhongqi Overpass.**

**Reply:** Thank you for your suggestion. In the revised manuscript, we have replaced “By using the calculation formula of the model established in this article and the correction formula for rust expansion and cracking width considering the influence of stirrups, the theoretical calculation value of steel corrosion rate can be calculated as 9.20%, and the actual detected steel corrosion rate is 10.56%, with a deviation of 12.88%.” with “The model was applied to the Sanhongqi Overpass, where the theoretical corrosion rate was calculated as 9.20%, compared to the measured value of 10.56%, with a deviation of 12.88%. This result confirms the model's applicability to real-world scenarios.”

**5. Provide recommendations for using the model in practical engineering.**

**Reply:** Thank you for your suggestion. In the revised manuscript, we have replaced “Therefore, based on comprehensive experimental research and on-site testing results, it is recommended to use a correction factor of 1.05 - 1.15 for theoretical calculation values according to actual engineering design parameters in practical engineering, which can meet the needs of rapid non-destructive testing technology in practical engineering.” with “To improve accuracy in practical applications, a correction factor between 1.05 and 1.15 is recommended, based on the specific design parameters of the structure, which can meet the needs of rapid non-destructive testing technology in practical engineering.”

**6. Acknowledge the limitations of the verification process.**

**Reply:** Thank you for your review and feedback on our manuscript. While the model shows high accuracy in predicting corrosion rates, factors such as environmental variability and external stresses were not considered and should be addressed in future studies.

**7. Use charts and graphs effectively.**

**Reply:** Thank you for your feedback. We have added a deviation analysis between the experimental values and the calculated values of the model, as shown in Table 3.

**Table 3. Analysis of the model calculated values and test data values of I# slab and II# slab**

| I# slab | | | | II# slab | | | |
| --- | --- | --- | --- | --- | --- | --- | --- |
| Rebar number | Measured value (%) | Calculated value (%) | Deviation  (%) | Rebar number | Measured value (%) | Calculated value (%) | Deviation  (%) |
| 1 | 1.63 | 1.52 | 6.75 | 7 | 0.53 | 0.48 | 9.43 |
| 2 | 1.63 | 1.50 | 7.98 |  |  |  |  |
|  |  |  |  | 8 | 0.55 | 0.49 | 10.91 |
| 3 | 1.66 | 1.56 | 6.02 |  |  |  |  |
| 4 | 1.26 | 1.11 | 11.90 | 9 | 0.60 | 0.53 | 11.67 |
| 5 | 0.79 | 0.72 | 8.86 |  |  |  |  |
|  |  |  |  | 10 | 0.70 | 0.64 | 8.57 |
| 6 | 1.66 | 1.54 | 7.23 |  |  |  |  |

Table 3, Figures 12 and 13 respectively show the comparison between the measured and calculated values of steel reinforcement corrosion in slabs I and II in the form of histograms. Comparison shows that for the case without distributed reinforcement, the minimum deviation of both is 6.02% and the maximum deviation is 11.90%, with deviations range from 6.0% to 12.0%; for the case with distributed reinforcement, the minimum deviation is 8.57% and the maximum deviation is 11.67%, with deviations range from 8.0% to 12.0%, it is indicated that the accuracy of the proposed model is relatively accurate which can effectively predict the amount of reinforcement corrosion. Considering the calculated value is smaller than the measured value in both cases, it is suggested to add a correction factor to the calculation model.

**8. Suggested Start of the Section.**

**Reply:** Thanks to your valuable suggestions. The proposed model was verified through two approaches: comparison with experimental data and application to a real-world engineering case. The experimental results confirmed the model's accuracy within an acceptable deviation range, while the practical application demonstrated its relevance to engineering practice.

**A structured presentation of findings and implications in the results and conclusion sections.**

**1. Start with a concise summary of the main findings from the study.**

**Reply:** Thank you for your valuable suggestion. In the revised manuscript, we have replaced “In this paper, an experimental and theoretical study on the relationship between the crack width of reinforced concrete slab as well as the amount of main reinforcement corrosion is carried out. The relationship between the amount of main reinforcement corrosion under the influence of distributed reinforcement and the crack width on the surface of the member is tested considering parameters such as concrete protective layer thickness and distributed reinforcement spacing.” with “This study presents a comprehensive analysis of the relationship between surface crack width and reinforcement corrosion in reinforced concrete slabs. By incorporating the influence of distributed reinforcement and protective layer thickness, the proposed model achieves high predictive accuracy, as confirmed by experimental and practical validation.”

**2. Clearly outline the contributions of the research.**

**Reply:** Thank you for your suggestion. The key contributions of this work include 1) Introducing a modified stiffness reduction factor for improved prediction accuracy, 2) Demonstrating the significant role of distributed reinforcement in reducing corrosion rates and crack propagation, and 3) Providing practical recommendations for optimizing structural durability in chloride environments.

**3. Relate the findings to practical engineering applications.**

**Reply:** Thank you for your advice. The proposed model offers a valuable tool for engineers to assess and mitigate corrosion in reinforced concrete structures, supporting proactive maintenance and extending service life.

**4. Briefly mention the study's limitations and areas for improvement.**

**Reply:** Thank you for your careful review and valuable feedback on our study's limitations and areas for improvement. While the model demonstrates high accuracy, factors such as environmental variability and the effects of external loads were not addressed and should be explored in future research.

**5. Suggest avenues for future research.**

**Reply:** Thank you for your suggestion. Future studies could focus on integrating additional variables, such as long-term environmental effects and multi-directional stress states, to further enhance the model's applicability.

**6. Suggested Start for the Conclusion.**

**Reply:** Thanks to your valuable suggestions. This study presents a comprehensive analysis of the relationship between surface crack width and reinforcement corrosion in reinforced concrete slabs. By incorporating the influence of distributed reinforcement and protective layer thickness, the proposed model achieves high predictive accuracy, as confirmed by experimental and practical validation.

**7. Conclude with a statement on the broader impact of the research.**

**Reply:** Thank you for your rigorous reviewing attitude. These findings contribute to advancing non-destructive testing techniques and improving the design and maintenance of reinforced concrete structures, addressing critical challenges in modern civil engineering.

Responses to Reviewer 2

Thank you for reviewing our manuscript and for your valuable comments. We take these suggestions very seriously and have revised the manuscript accordingly based on your feedback. The following is our itemized response to the review comments.

**1. The literature review should be more up-to-date. Papers published after 2023 are recommended.**

**Reply:** Thanks to your valuable suggestions. The Reference part has been updated, all papers cited are published in the last five years, and more than 50% of the papers were published after 2023.

[1] Niu D T. Durability of concrete in complex environments. Beijing: Science Press, 2020.

[2] Wang Q H, Zhou X, Sun X F, et al. Corrosion of Reinforcement in Reinforced Concrete: Influencing Factors, Detection Methods, and Control Techniques- A Review. Journal of Testing and Evaluation, 2024, 52(5): 3096-3122.

[3] Meyer YA, Menezes I, Bonatti RS, et al. EIS Investigation of the Corrosion Behavior of Steel Bars Embedded into Modified Concretes with Eggshell Contents. Metals. 2022; 12(3):417.

[4] Fu C, Huang J H, Dong Z, et al. Experimental and numerical study of an electromagnetic sensor for non-destructive evaluation of steel corrosion in concrete.Sensors and Actuators A: Physical, 2020, 315: 112371.

[5] Fang X, Yin Z D, Chen H X, et al. Comparative study on durability monitoring and testing of reinforced concrete structures in marine environment. Marine Engineering, 2021, 39(06): 111-118.

[6] Verstrynge E, Van Steen C, Vandecruys E, et al. Steel corrosion damage monitoring in reinforced concrete structures with the acoustic emission technique: A review. Construction and Building Materials, 2022, 349: 128732.

[7] Tian Y, Zhang G, Ye H, et al. Corrosion of steel rebar in concrete induced by chloride ions under natural environments. Construction and Building Materials, 2023, 369: 130504.

[8] Zhang X H, Wang H J, Zhang Y, et al. Corrosion of steel rebars across UHPC joint interface under chloride attack. Construction and Building Materials, 2023, 387(17): 131591.

[9] Fang X R, Pan Z C, Ma R J, et al. A multi-phase multi-species phase field model for non-uniform corrosion and chloride removal in concrete. Journal of Building Engineering, 2024, 82(1): 108214.

[10] Yan X R, Tian Y, Yan D M, et al. Corrosion-Induced Cracking Model of Concrete Considering a Transverse Constraint. Materials, 2024; 17(13): 3217.

[11] Zhu L, Zhang X H, Li D, et al. An experimental and numerical study on the corrosion characteristics of weathering steel-concrete composite beams. Construction and Building Materials, 2025, 462(7): 139893.

**2. The introduction should provide information that is more focused on the problem that the authors want to answer.**

**Reply:** Thank you very much for your suggestions. In the revised version, we have added more information that is more focused on the problem that the authors want to answer to strengthen the academic support and context of the paper. While significant progress has been made in understanding the relationship between corrosion and crack propagation in concrete structures, existing models[12-17] often neglect the influence of distributed reinforcement, which plays a crucial role in practical scenarios. This gap limits the applicability of these models in realworld conditions. The key contributions of this study include: 1) developing a novel calculation model that accounts for distributed reinforcement, 2) conducting experimental validation to ensure the model's practical reliability, and 3) providing insights for non-destructive testing methods applicable to real-world engineering.

**3. The figures used in the paper are not of good quality and do not speak for themselves.**

**Reply:** Thank you for your feedback. We have added a deviation analysis between the experimental values and the calculated values of the model, as shown in Table 3.

**Table 3. Analysis of the model calculated values and test data values of I# slab and II# slab**

| I# slab | | | | II# slab | | | |
| --- | --- | --- | --- | --- | --- | --- | --- |
| Rebar number | Measured value (%) | Calculated value (%) | Deviation  (%) | Rebar number | Measured value (%) | Calculated value (%) | Deviation  (%) |
| 1 | 1.63 | 1.52 | 6.75 | 7 | 0.53 | 0.48 | 9.43 |
| 2 | 1.63 | 1.50 | 7.98 |  |  |  |  |
|  |  |  |  | 8 | 0.55 | 0.49 | 10.91 |
| 3 | 1.66 | 1.56 | 6.02 |  |  |  |  |
| 4 | 1.26 | 1.11 | 11.90 | 9 | 0.60 | 0.53 | 11.67 |
| 5 | 0.79 | 0.72 | 8.86 |  |  |  |  |
|  |  |  |  | 10 | 0.70 | 0.64 | 8.57 |
| 6 | 1.66 | 1.54 | 7.23 |  |  |  |  |

Table 3, Figures 12 and 13 respectively show the comparison between the measured and calculated values of steel reinforcement corrosion in slabs I and II in the form of histograms. Comparison shows that for the case without distributed reinforcement, the minimum deviation of both is 6.02% and the maximum deviation is 11.90%, with deviations range from 6.0% to 12.0%; for the case with distributed reinforcement, the minimum deviation is 8.57% and the maximum deviation is 11.67%, with deviations range from 8.0% to 12.0%, it is indicated that the accuracy of the proposed model is relatively accurate which can effectively predict the amount of reinforcement corrosion. Considering the calculated value is smaller than the measured value in both cases, it is suggested to add a correction factor to the calculation model.

**4. Tables and figures should contain information about the statistical analysis, such as error bars.**

**Reply:** Thank you for your suggestion. Due to our experimental design of two reinforced concrete slabs with two types of steel corrosion forms and 10 specific steel bar samples, the discreteness of our experimental data is not significant. Therefore, there is no need to use statistical methods to process the experimental data. Instead, we will list all the experimental data in order to display the test results.

**5. It is recommended that the conclusion be written in a continuous text, not in items.**

**Reply:** We agree with the reviewer. The Conclusion section has been revised as follows: This study presents a comprehensive analysis of the relationship between surface crack width and reinforcement corrosion in reinforced concrete slabs. By incorporating the influence of distributed reinforcement and protective layer thickness, the proposed model achieves high predictive accuracy, as confirmed by experimental and practical validation. The proposed model offers a valuable tool for engineers to assess and mitigate corrosion in reinforced concrete structures, supporting proactive maintenance and extending service life. In addition, the corresponding semi-theoretical and semi-empirical model is given based on the existing research results, as well as the theoretical calculated values are compared with the experimental values. The key contributions of this work include 1) Introducing a modified stiffness reduction factor for improved prediction accuracy, 2) Demonstrating the significant role of distributed reinforcement in reducing corrosion rates and crack propagation, and 3) Providing practical recommendations for optimizing structural durability in chloride environments. The conclusions are as follows：

For the concrete slab without distributed reinforcement, the reinforcement in the specimens can be regarded as uniformly corroded with the accelerated rusting test by electrification. After cracking of the direction, the cracking width along the reinforcement is relatively uniform in the whole length direction, and the width of the cracks along the reinforcement basically does not differ much. As the thickness of the protective layer is larger, the corresponding amount of reinforcement corrosion and the width of surface cracks are smaller. For the concrete slab with distributed reinforcement, due to the inconsistent spacing of distributed reinforcement, after cracking of the specimen, the cracking width along the reinforcement is not uniform in the length direction, and as the spacing of distributed reinforcement is larger, the corresponding amount of reinforcement corrosion and the width of surface cracks is also larger.The theoretical formula given in this paper is small than the actual detection value, in order to make the prediction results more accurate and improve the detection efficiency, the calculation model of this paper can be corrected and the correction coefficient value range can be taken as 1.05 ~ 1.15.

While the model demonstrates high accuracy, factors such as environmental variability and the effects of external loads were not addressed and should be explored in future research. Future studies could focus on integrating additional variables, such as long-term environmental effects and multi-directional stress states, to further enhance the model's applicability. These findings contribute to advancing non-destructive testing techniques and improving the design and maintenance of reinforced concrete structures, addressing critical challenges in modern civil engineering.
